# Towards abundant intelligences: Considerations for Indigenous perspectives in adopting artificial intelligence technology

**DOI:** 10.1177/08404704241257144

**Published:** 2024-06-03

**Authors:** Julia A. Silano

**Affiliations:** 1 120481 Toronto Metropolitan University, Toronto, Ontario, Canada.

## Abstract

Artificial Intelligence (AI) applications in healthcare are evolving rapidly. The integration of AI into the Canadian healthcare system has demonstrated significant potential for enhancing the efficiency of care and improving patient outcomes. However, as this transformative technology continues to advance, it is crucial to take into account the unique perspectives and requirements of Indigenous Peoples in Canada. This article delves into the political, ethical, and practical considerations associated with introducing AI into Indigenous healthcare, emphasizing the paramount importance of equity and inclusion, which are rooted in the Two-Eyed AI framework. It also underscores the significance of co-creating AI technology in collaboration with Indigenous communities and multidisciplinary development teams. To illustrate these principles, this article spotlights an international AI epistemology-focused working group example. Healthcare professionals who engage with AI, whether it be through research, management, development, or leadership are implicated with this contemporary paradigm shift in decolonizing novel AI technology.

## Introduction

Artificial Intelligence (AI) in healthcare encompasses the application of advanced technologies and computer algorithms that simulate human intelligence and decision-making processes. This technology is utilized for the analysis of medical data and aims to support healthcare professionals in accurately diagnosing, treating, and predicting patient health outcomes.^1^ The transformative potential of AI in healthcare is revolutionary. In Indigenous communities, for example, AI technology aids in overcoming geographical challenges, enabling care providers and patients access to advanced diagnostics and expert medical insights that can be harder to obtain in under resourced and remote healthcare facilities.^2^ There are, however, ethical considerations surrounding AI and Indigenous Peoples within the Canadian healthcare context that revolve around multiple facets, such as cultural sensitivity, the incorporation of Indigenous Knowledge (IK) and values, equitable access, data sovereignty and governance, community engagement, and informed consent.^3^ These complex ethical concerns are rooted in Canada’s historical legacy of colonialism, intertwined with unethical medical research studies, that have been harmful to Indigenous communities specifically in the contexts of Western medicine and healthcare delivery.^4^

As Canada grapples with the Indigenous Rights Movement and endeavours towards reconciliation, it is imperative to prioritize efforts aimed at rectifying past shortcomings in the current implementation of AI policies within the healthcare sector.^5^ In 2017, Canada became the first country globally to launch a national research strategy backed by the Canadian Institute for Advanced Research (CIFAR).^6^ The Canadian government has a unique opportunity to incorporate Indigenous perspectives into AI development, in line with its obligations under Section 35 of the Canadian Constitution^7^ and commitment to the United Nations Declaration on the Rights of Indigenous Peoples (UNDRIP).^8^ While AI holds great promise for enhancing healthcare service efficiency, the absence of adequate consideration for Indigenous perspectives of health and well-being in the creation of such technology may result in healthcare technologies that are not well-suited to the specific challenges and socio-cultural determinants of health Indigenous populations face.^9^ Furthermore, AI can be enriched by Indigenous worldviews through the practice of Two-Eyed Seeing,^10^ which balances Indigenous and Western scientific viewpoints.

### The Silicon Valley doctrine

The composition of the technology workforce dominated by affluent male engineers of European descent influences the development of AI technology.^11^ The Silicon Valley perspective refers to a set of values, attitudes, and approaches to technology, entrepreneurship, and innovation that have emerged and become associated with a renowned tech innovation region in California.^5^ The Silicon Valley doctrine embodies a Western perspective that regards both humans and non-humans as exploitable resources,^12^ a perspective deeply intertwined with the legacies of colonization, capitalism, and slavery.^13^ Moreover, humans may need to develop an understanding of the ethical considerations or algorithmic biases that AI technologies may endow from their creators. Algorithmic bias has been defined as the application of algorithms exacerbating existing inequities in socioeconomic status, race, ethnicity, religion, gender, disability, or sexual orientation, particularly within health systems.^14,15^ For example, algorithms employed by prominent health systems have exhibited racial bias for marginalized populations, including Black,^16^ LGBTQ2S+, Asian,^17^ and Indigenous populations.^18^ Bias and underrepresentation in the creation of AI technologies have a trickle-down effect, exacerbating social inequalities and potentially causing patient harm through the misinterpretation of personal health data.^19^ Ultimately, the peril in Silicon Valley may lie in its exclusive incorporation of the dominant Western worldview in the development of conscious machines.

### The breath of AI

Kinship-based knowledge systems emphasize the collective, holistic, and relational aspects of Indigenous ways of knowing to promote human flourishing.^20^ This belief is core to many Indigenous epistemologies. If Western philosophical and scientific traditions lack “relational” understanding of humanity’s place in the world, conversely, many Indigenous cultures place importance on relationships with inanimate and animate objects.^20,21^ For example, in Lakota ontologies, non-human relationships are prioritized, especially those with stones.^22^ In essence, non-humans are viewed as having volition and decision-making.^23^ These epistemologies form the foundation for ways of knowing and communicating that recognize the interconnected relationships that extend to wildlife, plants, the elements, water, and land.^24^ Similarly, AI is an extension of personhood to an inanimate object such as a technological device, software code or algorithm.^21^ It is essential to highlight that there is no singular Indigenous epistemology. This article will use the terms *Indigenous epistemology* and *perspective* to describe frameworks originating from Indigenous nations in North America and the Pacific Ocean, which hold commonalities in their perspectives on human to non-human kin relationships.^22^ When considering the development of more ethical, sustainable, and culturally sensitive AI applications that can serve the well-being of both individuals and the broader ecosystem, ultimately contributing to a more balanced society.^25^

### Two-Eyed AI

Two-Eyed AI represents an AI approach that encompasses both IK and Western scientific perspectives. This concept of Two-Eyed AI is rooted in the Two-Eyed Seeing principle, championed by prominent Mi’kmaw Elders, Albert and Murdena Marshall.^19^ Two-Eyed Seeing entails the art of perceiving the strengths inherent in Indigenous wisdom balanced with the strengths found in Western knowledge and ways of understanding.^19^ This approach involves one eye focused on human ethics concerns and the other on recognizing the technological potential.^10,19^ For example, in Canada, AI is being used to help preserve polysynthetic Indigenous languages like Mohawk, Algonquin, and Michif through speech- and text-based applications.^26^ In essence, Two-Eyed AI is a practical manifestation of decolonization theory in the realm of AI. It promotes a seamless fusion of AI technologies with Indigenous culture, with the goal of empowering Indigenous communities in the digital era while preserving their culturally rich heritage.

### Case study: Co-creation of AI technology with Indigenous communities

#### Background

In 2019, the CIFAR released a position paper in conjunction with the Pan-Canadian AI Strategy, focusing on AI development through the lens of Indigenous perspectives.^27^ This document marked the inception of a more ethically oriented AI strategy called the Abundant Intelligences Project (AIP). Co-directed by Prof. J.E. Lewis (Concordia University, Quebec) and Prof. H. Whaanga (Massey University, New Zealand), the AIP aims to make AI more inclusive of Indigenous perspectives.^28^ This ongoing government funded project involves investigators and collaborators, primarily of Indigenous background, from universities and Indigenous community-based organizations across Canada, the United States, and New Zealand.^10^

#### Strengths of the abundant intelligences project

The three-pronged framework of the AIP project is a unique application of the Two-Eyed Seeing principle. The AIP explores three distinct axes, each addressing vital aspects of IK integration into mainstream AI applications. Integration, the first axis prioritizes active community engagement while merging IK practices and mainstream AI research frameworks. The establishment of physical spaces, “pods,” facilitates deep engagement within participating Indigenous communities.^27^ Indigenous communities recognize stakeholders as part of their extended network of relations, whereas technology companies typically identify stakeholders as comprising the board of directors, shareholders, employees, and consumers.^15^ This inclusive and collaborative process acknowledges that any co-created AI solutions respect both human and non-human entities.

The strength of the second axis, Imaginaries, lies in designing AI systems tailored to the specific needs and aspirations of Indigenous communities.^27^ By taking into account geographic isolation, linguistic diversity, and cultural traditions, it addresses the unique challenges faced by these communities. The incorporation of Lakȟóta design principles into AI wearable devices, for example, highlights, this approach’s innovation and adaptability.^29^ Moreover, including Indigenous imaginaries respects the fundamental principles of sovereignty and self-determination,^16^ ensuring that AI technology aligns with the community’s values and priorities.

Intelligence, the third axis, addresses technical challenges in mainstream AI research by applying Indigenous worldviews. By striving to translate the full spectrum of human intelligence into machine intelligence, the AIP ensures that AI technologies are developed and deployed with respect for and preservation of Indigenous cultures, languages, and traditions.^27^ For example, when AI design is approached from a Māori perspective, it accentuates the significance of hapū (“sub-tribes”) and whānau (“family”),^30^ underscoring the potential for more holistic and culturally resonant AI applications. Applying this principle to technologies like facial recognition, which often include images of both living and deceased individuals, breaches Tapu (“sacred”) principles.^18^ Therefore, AI development needs to be culturally sensitive, even challenging Western technocratic norms where cultural content may be seen as expendable for efficiency and optimization.^18^ This approach enriches AI research as it also promotes cultural preservation and inclusivity in technology development.

#### Limitations of the abundant intelligences project

AI has the potential to promote equity, but its positive impact is more likely when the development is undertaken by entities not primarily motivated by profit, such as hospitals or universities.^31^ The allocation of government funding can profoundly influence the trajectory and outcomes of the AIP. The pan-Canadian AI Strategy backing the CIFAR and the AIP, currently receives financial backing from major technology players such as Facebook, a commercial bank, plus government funds, thereby exemplifying a strong government-industry-academia collaboration.^6^

However, the 5-year, $125 million investment in the pan-Canadian AI Strategy pales in comparison to funding in other countries, subsequently prompting concerns about Canada’s historical challenges in commercializing research outcomes.^6^ There is a danger in AI development that developers may tokenistically apply these values without bringing about substantial changes in how AI is conceived and crafted.^32^ Highlighting this concern is the proposed Bill-C27, which concerns updating AI privacy laws, and the government’s criticism as “anti-democratic” for not consulting Indigenous leaders and relying too heavily on feedback from industry.^33^ Moreover, shrinking contemporary fixations of technological speed, scale, and innovation ought to be disrupted through Indigenous centred AI development.^34^ Perhaps only governmental and legislative drivers that may force policymakers and other influential technology sector parties to ground their goals around larger questions of equity, colonial legacies, and justice will suffice. The challenge of regulating the complex decisions for ethical AI will demand significant public engagement and scrutiny because the essentials for fair AI development go far beyond regulatory boundaries.^32^ In the end, a well-defined national policy is essential to delineate the parameters within which AI and Indigenous worldviews will collaboratively evolve in Canada.^20^

## Considerations for health leaders

### Abundance over singularity

“Abundant intelligences” encapsulates the notion of diverse and thriving knowledge practices within specific environments.^30^ Drawing on the AIP framework, health leaders and AI working groups may actively embrace strategies within their organizations to ensure a culturally sensitive and inclusive approach. The term “abundant” signifies not only the richness of these practices but also their potential to shape numerous futures and challenge narrow perceptions of intelligence.^35^ Health leaders who understand the importance of incorporating Indigenous worldviews into AI development are better positioned to manage culturally sensitive, equitable, and inclusive organizations. As emphasized by the 2015 TRC Calls to Action,^36^ the praxis of the AIP can guide healthcare leaders to ensure the inclusion, respect, and empowerment of Indigenous communities during this technological transformation. By lowering the predominant Western viewpoint and incorporating alternative paradigms, the more adept AI developers may become at achieving social and ethical objectives.

### Health leadership towards abundantly intelligent organizations

There is potential for infusing Indigenous worldviews into the integration of AI at multiple levels within healthcare organizations. At the development level, designers, engineers, and computer scientists can incorporate diverse perspectives to inform the creation process.^35^ This may involve developing adaptable, modular systems customized to fit specific contexts while maintaining core elements that respect Indigenous cultural identities, histories, and worldviews. Creating wider gender identification spectra to include Two Spirit is one example.^37^ At the management tier, tools such as end-to-end AI development frameworks that allow for recurring audits enable managers to better mediate the implementation of new technology in conjunction with Indigenous stakeholders.^38^ Such tools can enable development teams to assess how an AI model performs under various conditions, including its effectiveness across diverse human health datasets.^10,39^ Moreover, culturally diverse AI development teams are crucial for designing and developing inclusive AI applications.^40^ An emphasis should also be made on recruiting and retaining Indigenous talent in both healthcare and AI fields to further enhance the creation and uptake of abundantly intelligent software.

Providing cultural competency training for healthcare staff, including AI developers and users, enables more culturally sensitive care and the culturally appropriate use of AI technologies. Participatory exercises such as the “KAIROS Blanket Exercise” lead by Indigenous elders or educators promote truth, understanding, and reconciliation among both Indigenous and non-Indigenous communities alike.^41,42^ Creating shared spaces, coined “pods” by the AIP, whereby Indigenous community members facilitate deep learning and IK sharing practices, such as talking circles and smudge ceremonies reflect culturally sensitive approach.^29,43^

Establishing ethical oversight committees with Indigenous representation to review AI technologies in tandem with current and novel government policies can help to ensure better alignment with Indigenous ethical principles. Furthermore, sharing best practices and successful case studies related to integrating Indigenous perspectives into AI in healthcare contributes to interdisciplinary and cross-sector knowledge sharing.^42^ Without widespread understanding of the impacts of singular AI systems, healthcare equity challenges as seen in differential outcomes during the COVID-19 pandemic in Canada will persist.^39^

Although an exhaustive discussion of decolonization is beyond the scope of the article, this brief overview is a catalyst for practically merging Two-Eyed Seeing principles into healthcare organizational change strategies. At the heart of this discussion is the need for health leaders to honour the ethical imperative of, “nothing about us without us” through ongoing consultation with Indigenous Peoples.^44^ In the same vein, as health leaders understand the issues within AI systems, lines of accountability can become more clearly defined within the healthcare organizations that they serve.

## Conclusion

As AI advances, so should the integration of the distinct perspectives and needs of Indigenous Peoples. Principles deeply embedded in The Two-Eyed AI framework can guide the inclusion of Indigenous ethos and imaginaries into AI creation balanced with scientific and technological constructs.^27^ Moreover, as health leaders strive towards fostering collaboration with Indigenous Peoples, this AI, in turn, will aid in restructuring our societal landscapes, reshaping our perceptions of digital territories, and supporting individuals in acknowledging and respecting Indigenous viewpoints.^22^ By prioritizing Indigenous viewpoints in the advancement of AI and AI policy, exemplified through the federal government’s endorsement of the AIP, Canada can enhance its endeavours to foster ethical and inclusive AI.

## References

[1] Naik N Hameed BM Shetty DK et al. Legal and ethical consideration in artificial intelligence in healthcare: who takes responsibility? Front Surg. 2022;9:862322. 35360424 10.3389/fsurg.2022.862322 PMC8963864

[2] Darcel K Upshaw T Craig-Neil A , et al. Implementing artificial intelligence in Canadian primary care: barriers and strategies identified through a national deliberative dialogue. PLoS One. 2023;18(2):e0281733. doi:10.1371/journal.pone.0281733 36848339 PMC9970060

[3] Henderson B Flood C Scassa T . Number. Technology-Facilitated gender-based violence article 10 1-2022 Artificial intelligence in Canadian healthcare: will the law protect us from algorithmic bias resulting in discrimination? Canadian Journal of Law and Technology Canadian Journal of Law and Technology. 2022;19:2-475. https://digitalcommons.schulichlaw.dal.ca/cgi/viewcontent.cgi?article=1282&context=cjlt

[4] Kim PJ . Social determinants of health inequities in indigenous Canadians through a life course approach to colonialism and the residential school system. Health Equity. 2019;3(1):378-381. doi:10.1089/heq.2019.0041 31346558 PMC6657289

[5] Cook K . The psychology of silicon valley. Springer International Publishing; 2020;12-14. doi:10.1007/978-3-030-27364-4

[6] Brandusescu A . Artificial intelligence policy and funding in Canada: public investments, private interests. SSRN Journal. 2021:17-41. doi:10.2139/ssrn.4089932

[7] Government of Canada . Consolidated Federal Laws of Canada, the Constitution Acts, 1867 to 1982. Laws-Lois.justice.gc.ca. Published August 7, 2020. https://laws-lois.justice.gc.ca/eng/const/page-13.html

[8] Assembly, UN General . *United Nations declaration on the rights of indigenous* peoples. *UN Wash* . 2007. 12:1-18.

[9] Dubay L . Creating an equitable AI policy for Indigenous communities. First Policy Response. https://policyresponse.ca/creating-an-equitable-ai-policy-for-indigenous-communities/. Published 12 November 2021.

[10] Bourgeois-Doyle D . Two-Eyed AI: A Reflection on Artificial Intelligence. Paris: The Canadian Commission for UNESCO. United Nations Educational, Scientific and Cultural Organization; 2019.

[11] West S . DISCRIMINATING SYSTEMS gender, race, and power in AI. 2019. https://ainowinstitute.org/wp-content/uploads/2023/04/discriminatingsystems.pdf

[12] Jiménez A . The Silicon doctrine. tripleC. 2020;18(1):322-336. doi:10.31269/triplec.v18i1.1147

[13] Cheney J . Postmodern environmental ethics: ethics of bioregional narrative. Environ Ethics. 1989;11(2):117-134.

[14] Mittermaier M Raza MM Kvedar JC . Bias in AI-based models for medical applications: challenges and mitigation strategies. NPJ Digit Med. 2023;6(1):113. doi:10.1038/s41746-023-00858-z 37311802 PMC10264403

[15] Panch T Mattie H Atun R . Artificial intelligence and algorithmic bias: implications for health systems. J Glob Health. 2019;9(2):010318. doi:10.7189/jogh.09.020318 PMC6875681 31788229

[16] Obermeyer Z Powers B Vogeli C Mullainathan S . Dissecting racial bias in an algorithm used to manage the health of populations. Science. 2019;366(6464):447-453. doi:10.1126/science.aax2342 31649194

[17] Wong AKI Charpignon M Kim H , et al. Analysis of discrepancies between pulse oximetry and arterial oxygen saturation measurements by race and ethnicity and association with organ dysfunction and mortality. JAMA. 2021;4:e2131674. 10.1001/jamanetworkopen.2021.31674 PMC9178439 34730820

[18] Comeau D Johnson C Bouhamdani N . Review of current 2SLGBTQIA+ inequities in the Canadian health care system. Front Public Health. 2023;11:1183284. doi:10.3389/fpubh.2023.1183284 37533535 PMC10392841

[19] Igoe K . Algorithmic bias in health care exacerbates social inequities — how to prevent it. Executive and Continuing Professional Education, Harvard T.H. Chan School of Public Health. 2021. https://www.hsph.harvard.edu/ecpe/how-to-prevent-algorithmic-bias-in-health-care/

[20] Lewis JE . Indigenous protocol and artificial intelligence workshops position paper. Indigenous Protocol and Artificial Intelligence Indigenous Protocol and Artificial Intelligence Working Group Honolulu, Hawai’i. Montreal, QC: Concordia University; 2020. doi:10.11573/spectrum.library.concordia.ca.00986506

[21] McMaster G . Creating ethical AI from Indigenous perspectives. www.ualberta.ca https://www.ualberta.ca/folio/2020/10/creating-ethical-ai-from-indigenous-perspectives.html

[22] Lewis JE Arista N Pechawis A Kite S . Making kin with the machines. J Des Sci. 2018:4-10. doi:10.21428/bfafd97b

[23] Kesserman K . How can Indigenous knowledge shape our view of AI? Policy Options. https://policyoptions.irpp.org/magazines/february-2018/how-can-indigenous-knowledge-shape-our-view-of-ai/. Published online 16 February 2018.

[24] Little Bear L . Naturalizing Indigenous Knowledge Synthesis Paper; 2009. https://neatoeco.com/iwiseconference.org/wp-content/uploads/2015/08/NaturalizingIndigenousKnowledge_LeroyLittlebear.pdf

[25] Stahl BC . Responsible innovation ecosystems: ethical implications of the application of the ecosystem concept to artificial intelligence. Int J Inf Manag. 2022;62:102441. doi:10.1016/j.ijinfomgt.2021.102441.

[26] Iwama M Marshall M Marshall A , et al. Two-eyed seeing and the language of healing in community-based research. Can J Native Educ. 2009;32(2). doi:10.14288/cjne.v32i2.196493

[27] Barron B Chowdhury N Davidson K , et al. Annual Report of the CIFAR Pan-Canadian AI Strategy; 2019. https://cifar.ca/wp-content/uploads/2020/04/ai_annualreport2019_web.pdf. Accessed 17 April 2024.

[28] INDIGENOUS AI . Abundant Intelligences; 2019. https://www.indigenous-ai.net/abundant/. Accessed 17 April 2024.

[29] New Frontiers Research Fund . Indigenous-Led AI: How Indigenous Knowledge Systems Could Push AI to Be More Inclusive. Government of Canada; 2023. www.sshrc-crsh.gc.ca. https://www.sshrc-crsh.gc.ca/funding-financement/nfrf-fnfr/stories-histoires/2023/inclusive_artificial_intelligence-intelligence_artificielle_inclusive-eng.aspx

[30] Selwyn N . Designing and Evaluating AI According to Indigenous Māori Principles (Notes on Munn 2023). Critical Studies of EDUCATION & TECHNOLOGY. Published June 16, 2023. https://criticaledtech.com/2023/06/16/munn-l-2023-the-five-tests-designing-and-evaluating-ai-according-to-indigenous-maori-principles-ai-society/. Accessed 20 April 2024.

[31] Glauser W . AI in health care: improving outcomes or threatening equity? Can Med Assoc J. 2020;192(1):E21-E22. doi:10.1503/cmaj.1095838 31907237 PMC6944301

[32] Munn L . The uselessness of AI ethics. AI Ethics. 2022;3(3):869-877. doi:10.1007/s43681-022-00209-w

[33] News CTV . First Nations, Jim Balsillie slam government over lack of consultation on AI bill. CTVNews. 2024. https://www.ctvnews.ca/politics/first-nations-jim-balsillie-slam-government-over-lack-of-consultation-on-ai-bill-1.6769402. Accessed 20 April 2024.

[34] Munn L . The five tests: designing and evaluating AI according to indigenous Māori principles. AI & Society; 2023:6-9. doi:10.1007/s00146-023-01636-x

[35] Durand C . OCAD U Participates in Research Project on How Indigenous Knowledges Can Improve AI for All. OCAD University. https://www.ocadu.ca/news/ocad-u-participates-research-project-how-indigenous-knowledges-can-improve-ai-all. Accessed 20 April 2024.

[36] Truth and Reconciliation Commission of Canada . Truth and Reconciliation Commission of Canada: Calls to Action. Truth and Reconciliation Commission of Canada; 2015. https://www2.gov.bc.ca/assets/gov/british-columbians-our-governments/indigenous-people/aboriginal-peoples-documents/calls_to_action_english2.pdf

[37] I Scheim A Jackson R James L Sharp Dopler T Pyne J R Bauer G . Barriers to well-being for aboriginal gender-diverse people: results from the trans PULSE project in Ontario, Canada. Ethnicity and Inequalities in Health and Social Care. 2013;6(4):108-120. doi:10.1108/eihsc-08-2013-0010

[38] Raji ID Smart A White RN , et al. Closing the AI accountability gap. Proceedings of the 2020 Conference on Fairness, Accountability, and Transparency. New York, NY: Association for Computing Machinery. doi:10.1145/3351095.3372873

[39] Thomasian NM Eickhoff C Adashi EY . Advancing health equity with artificial intelligence. J Publ Health Pol. 2021;42(4):602-611. doi:10.1057/s41271-021-00319-5 PMC8607970 34811466

[40] Shams RA Zowghi D Bano M . AI and the quest for diversity and inclusion: a systematic literature review. AI Ethics. 2023:4. doi:10.1007/s43681-023-00362-w

[41] Herzog LS Wright SR Pennington JJ Richardson L . The KAIROS blanket exercise: engaging Indigenous ways of knowing to foster critical consciousness in medical education. Med Teach. 2021;43:1437-1443. doi:10.1080/0142159x.2021.1956679 34369238

[42] Health Care Excellence Canada . IMPLEMENTING ARTIFICIAL INTELLIGENCE in CANADIAN HEALTHCARE: A KIT for GETTING STARTED Principles and Guidance from an Expert Design Lab Process Executive Summary and Report; 2021. https://www.healthcareexcellence.ca/media/qdmn0p3b/20211208_aireport_en.pdf

[43] Hunt SC Young NL . Blending indigenous sharing circle and western focus group methodologies for the study of indigenous children’s health: a systematic review. Int J Qual Methods. 2021;20:160940692110151. doi:10.1177/16094069211015112

[44] McGibbon E . Truth and reconciliation: healthcare organizational leadership. Healthc Manag Forum. 2018;32(1):20-24. doi:10.1177/0840470418803379 30514125

